# Connexins and Pannexins: New Insights into Microglial Functions and Dysfunctions

**DOI:** 10.3389/fnmol.2016.00086

**Published:** 2016-09-22

**Authors:** Rosario Gajardo-Gómez, Valeria C. Labra, Juan A. Orellana

**Affiliations:** Departamento de Neurología, Escuela de Medicina, Pontificia Universidad Católica de ChileSantiago, Chile

**Keywords:** microglia, hemichannels, gap junctions, pannexons, brain, gliotransmitters

## Abstract

Under physiological conditions, microglia adopt a resting phenotype associated with the production of anti-inflammatory and neurotrophic factors. In response to a wide variety of insults, these cells shift to an activated phenotype that is necessary for the proper restoration of brain homeostasis. However, when the intensity of a threat is relatively high, microglial activation worsens the progression of damage rather than providing protection, with potentially significant consequences for neuronal survival. Coordinated interactions among microglia and other brain cells, including astrocytes and neurons, are critical for the development of timely and optimal inflammatory responses in the brain parenchyma. Tissue synchronization is in part mediated by connexins and pannexins, which are protein families that form different plasma membrane channels to communicate with neighboring cells. Gap junction channels (which are exclusively formed by connexins in vertebrates) connect the cytoplasm of contacting cells to coordinate electrical and metabolic coupling. Hemichannels (HCs) and pannexons (which are formed by connexins and pannexins, respectively) communicate the intra- and extracellular compartments and serve as diffusion pathways for the exchange of ions and small molecules. In this review article, we discuss the available evidence concerning the functional expression and regulation of connexin- and pannexin-based channels in microglia and their contributions to microglial function and dysfunction. Specifically, we focus on the possible implications of these channels in microglia-to-microglia, microglia-to-astrocyte and neuron-to-microglia interactions in the inflamed brain.

## Background

Microglia constitute approximately 5–15% of total cells in the central nervous system (CNS) and are the major components of the innate immune system in the brain (Lawson et al., 1990). Originating from myelomonocytic precursor cells (i.e., fetal macrophages) derived from the hemangioblastic mesoderm, microglia populate the brain parenchyma prior to the developmental closure of the blood-brain barrier (BBB; Ginhoux et al., 2010). Under physiological conditions, microglia exhibit a “resting” surveillance state (ramified morphology) that is associated with active environmental exploration and the continuous search for exogenous or endogenous signals representing a brain threat (Streit, 2001; Kettenmann et al., 2011). Microglia assume protagonistic roles that extend far beyond their classic participation in inflammatory and immunological responses, such as the control of neuronal proliferation and differentiation and synaptic plasticity and transmission (Wu et al., 2015). In particular, the fine interactions between microglia and chemical synapses are essential for the consolidation of neuronal circuits, thereby expanding the current concept of the “tripartite synapse” into a “quad-partite synapse” (Schafer et al., 2013; Wake et al., 2013).

Once homeostatic balance is altered, the resting microglia phenotype shifts to a reactive phenotype (phagocytic morphology) involving alterations to different microglial functions, such as proliferation, morphology, motility and migration, proteostasis, phagocytosis and intercellular communication (Hanisch, 2002; Block et al., 2007). This wide array of changes is known as “microglial activation” and involves large-scale and substantial activation depending on the nature, intensity and duration of the stimulus (Ransohoff and El Khoury, 2015). During intense challenges and chronic brain damage, microglia become uncontrolled sources of inflammatory mediators (e.g., cytokines and free radicals) that cause neuronal damage rather than exhibiting a repair-orientated activity profile (Block et al., 2007). Although an efficient immune response is necessary to resolve brain threats, under these circumstances, dysfunctional microglia favor the recruitment of non-resident brain cells involved in the innate and adaptive immune responses, thereby intensifying brain injury (Waisman et al., 2015).

## General Concepts Concerning Connexins and Pannexins

**Figure 1:**
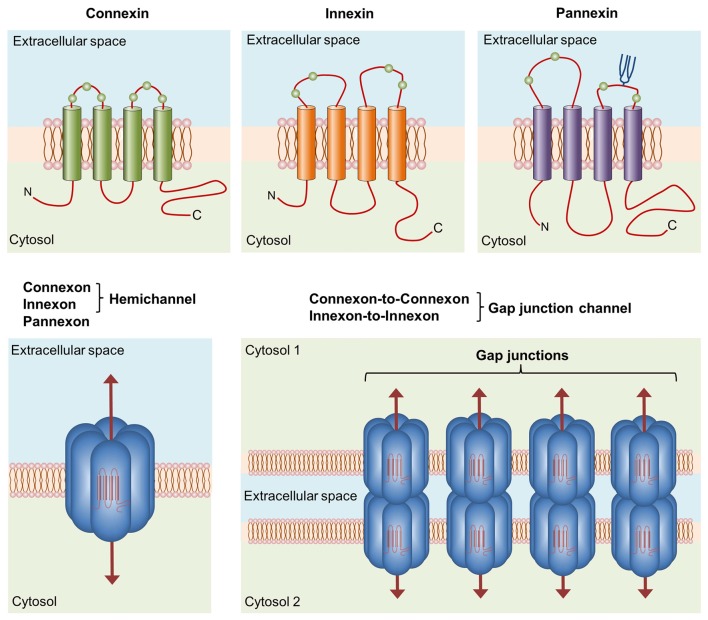
**Basic structure of channels formed by connexins, innexins and pannexins.** Connexins, innexins and pannexins share similar membrane topologies consisting of four α-helical transmembrane domains connected by two extracellular loops and one cytoplasmic loop; both the amino- and carboxy-termini are intracellular. The relative positions of the extracellular loop cysteines (green balls) and glycosylated asparagines (blue branches) are also shown. Connexons or innexons (also known as hemichannels (HCs)) are formed from connexins or innexins consisting of six subunits each. Pannexons are single membrane channels composed of six pannexin subunits. Recently, a Panx2 band pattern more consistent with an octamer than a hexamer was observed in cross-linking studies and native gels examining purified homomeric full-length and C-terminal truncation mutants (Ambrosi et al., 2010). Under resting conditions, HCs and pannexons remain preferentially closed, but they are activated by diverse physiological and pathological conditions and represent a diffuse transmembrane route between the intra- and extracellular milieu. HCs dock with one another to form functional cell-to-cell channels termed gap junction channels (GJCs). GJCs aggregate in well-known anatomical structures called gap junctions to facilitate the intercellular exchange of metabolites, second messengers and ions.

Transitions and commitments towards distinct reactive phenotypes require close synchronization and communication among microglia (Salter and Beggs, 2014). In vertebrates, cell-to-cell communication and coordination between neighboring cells are in part mediated by gap junctions (Bennett et al., 1991; Sáez et al., 2003). Gap junctions are aggregates of intercellular channels termed gap junction channels (GJCs) that facilitate direct but selective cytoplasmic continuity between contacting cells to promote the exchange of ions (allowing electrical coupling), metabolites (e.g., adenosine diphosphate [ADP], glucose, lactate and glutamate) and second messengers (e.g., cyclic adenosine monophosphate [cAMP] and inositol trisphosphate [IP_3_]; Söhl et al., 2005; Goodenough and Paul, 2009; Figure 1). A GJC comprises the serial docking of two hemichannels (HCs), one of each contributed by two adjacent cells. Each HC consists of a six-fold ring of connexin monomers; thus, these channels are also known as connexons (Figure 1). Connexins are members of a highly conserved protein family encoded by 21 genes in humans and 20 genes in mice with orthologs in other vertebrate species (Abascal and Zardoya, 2012).

Although they have long been recognized as the building blocks of GJCs, HCs are fundamental pathways for molecular and ionic interchange between intracellular and extracellular compartments (Sáez and Leybaert, 2014). In a pioneering study by Paul et al. (1991), cytoplasmic injection of *Xenopus* oocytes with connexin46 (Cx46) mRNA (a newly cloned member of the connexin family at the time) caused the induction of non-selective cation currents, depolarization and cell lysis in single oocytes. This phenomenon was not observed after transfection with other connexins, leading to the following rationale: open HCs disturb ionic imbalance, resulting in the persistent loss of membrane potential and subsequent cell death. Therefore, HC openings were assumed to be incompatible with normal cell life. However, this concept has been disproven in the last two decades by groundbreaking findings (Cotrina et al., 1998; Stout et al., 2002; Ye et al., 2003; Cherian et al., 2005; Romanov et al., 2007; Tong et al., 2007; Anselmi et al., 2008; Burra et al., 2010; Garré et al., 2010; Orellana et al., 2012a; Stehberg et al., 2012; Chever et al., 2014; Roux et al., 2015). According to current thinking, the physiological opening of HCs is required for the release of paracrine substances into the extracellular milieu (e.g., adenosine triphosphate [ATP], glutamate, nicotinamide adenine dinucleotide [NAD^+^] and prostaglandin E2 [PGE_2_]) and the uptake of small molecules and ions up to ~1–1.2 kDa in size (e.g., glucose, cyclic ADP-ribose [cADPR] and Ca^2+^; Wang et al., 2013; Sáez and Leybaert, 2014; Figure 1).

A family of chordate proteins homologous to the “innexins” (the gap junction proteins of invertebrates) was discovered more than a decade ago (Panchin et al., 2000). This three-member family was designated the “pannexins” (Panx1–3) due to their ubiquitous presence in all eumetazoans with the exception of echinoderms (Abascal and Zardoya, 2012; Bond and Naus, 2014; Figure 1). Although pannexins form GJCs when overexpressed in *Xenopus* oocytes (Bruzzone et al., 2003), it seems that pannexins lose their ability to directly couple adjacent cells *in vivo* (Sosinsky et al., 2011). The major function of pannexins is form single membrane channels (the so-called pannexons) similar to HCs that permit paracrine/autocrine signaling with specific features among cells (Dahl, 2015).

In the nervous system, HCs and pannexons fulfill important physiological functions, including ischemic tolerance (Lin et al., 2008; Schock et al., 2008), the establishment of adhesive interactions (Cotrina et al., 2008), fear memory consolidation (Stehberg et al., 2012), synaptic transmission (Klaassen et al., 2011; Prochnow et al., 2012; Ardiles et al., 2014; Chever et al., 2014), electrical activity and oscillations (Moore et al., 2014; Lopatář et al., 2015; Roux et al., 2015), glucose sensing (Orellana et al., 2012a), mast cell degranulation (Harcha et al., 2015), chemoreception (Wenker et al., 2012), BBB permeability (De Bock et al., 2011; Kaneko et al., 2015), colonic transit (McClain et al., 2014), migration (Liu et al., 2010, 2012) and proliferation (Wicki-Stordeur et al., 2012). In contrast, different pathological scenarios modify the normal functions of HCs and pannexons, thereby altering their opening and permeability properties and contributing to the onset and progression of diverse brain diseases (Orellana et al., 2012b, 2013a; Bosch and Kielian, 2014; Shestopalov and Slepak, 2014; Takeuchi and Suzumura, 2014; Decrock et al., 2015). Here, we review and discuss the available evidence concerning the functional expression and regulation of GJCs, HCs and pannexons in microglia and their contributions to microglial function and dysfunction (Tables 1, 2).

**Table 1:** **Regulation of connexin and pannexin expression in microglia**.

Effector	Protein levels	mRNA levels	Technique	Reference
Cryotraumatic brain injury	↑Cx29↑Cx32	NT	Immunohistochemistry	Moon et al. (2010)
LPS	↑Cx32	NT	Flow cytochemistry	Takeuchi et al. (2006)
MeCP2 deficiency	↑Cx32	NE	RT-PCR, Immunohistochemistry, Western blotting	Maezawa and Jin (2010)
TNF-α	↑Cx32	NT	Flow cytochemistry	Takeuchi et al. (2006)
GM-CSF	NE Cx36	NT	Western blotting	Dobrenis et al. (2005)
IFN-γ + TNF-α	↑Cx36	NT	Western blotting	Dobrenis et al. (2005)
LPS	NT	NE Cx36	RT-PCR	Dobrenis et al. (2005)
Rasmussen encephalitis	↑Cx36	NT	Immunohistochemistry	Cepeda et al. (2015)
AGEs	↑Cx43	NT	Western blotting	Shaikh et al. (2012)
Amyloid β-peptide	↑Cx43	NT	Western blotting	Orellana et al. (2011b)
Brain stab wound	↑Cx43	NT	Immunocytochemistry	Eugenín et al. (2001)
Calcium ionophore	↑Cx43	↑Cx43	Immunohistochemistry, Western blotting, RT-PCR	Martínez et al. (2002)
Glioma	NT	NF Cx43	RT-PCR	Richter et al. (2014)
GM-CSF	NF Cx43	NT	Western blotting	Dobrenis et al. (2005)
HIV encephalitis	↑Cx43	NT	Immunocytochemistry	Eugenin et al. (2012)
LPS	NF Cx43	NT	Western blotting, Immunohistochemistry	Même et al. (2006)
IFN-γ + TNF-α	NF Cx43	NT	Western blotting	Dobrenis et al. (2005)
IFN-γ + TNF-α	↑Cx43	NT	Immunocytochemistry, Western blotting	Eugenín et al. (2001)
Peptidoglycan	↑Cx43	↑Cx43	RT-PCR, Western blotting	Garg et al. (2005)
Spinal cord injury	NE Cx43	NT	Immunohistochemistry	Lee et al. (2005)
Restraint stress	NE Cx43	NT	Immunohistochemistry	Orellana et al. (2015)
TNF-α	↑Cx43	NT	Western blotting	Shaikh et al. (2012)
TNF-α + ATP	↑Cx43	NT	Western blotting	Sáez et al. (2013)
TNF-α + IL-1β	↑Cx43	NT	Western blotting	Sáez et al. (2013)
TNF-α + IFN-γ	↑Cx43	NT	Western blotting	Sáez et al. (2013)
Transplantation of NPCs	↑Cx43	NT	Immunohistochemistry	Talaverón et al. (2014)
Amyloid β-peptide	↑Panx1	NT	Western blotting	Orellana et al. (2011b)
IFN-γ	NT	↑Panx1	RT-PCR	Shestopalov and Slepak (2014)
Prenatal nicotine and postnatal HFC diet	NE Panx1	NT	Immunohistochemistry	Orellana et al. (2014)
Restraint stress	NE Panx1	NT	Immunohistochemistry	Orellana et al. (2015)
Rasmussen encephalitis	↑Panx1	NT	Immunohistochemistry	Cepeda et al. (2015)
TNF-α + ATP	↑Panx1	NT	Western blotting	Sáez et al. (2013)
TNF-α + IL-1β	↑Panx1	NT	Western blotting	Sáez et al. (2013)
TNF-α + IFN-γ	↑Panx1	NT	Western blotting	Sáez et al. (2013)

*NE, no effect; NT, not tested; NF, not found; LPS, lipopolysaccharide; Mecp2, methyl-CpG binding protein 2; TNF-α, tumor necrosis factor alpha; GM-CSF, granulocyte-macrophage colony-stimulating factor; IFN-γ, interferon gamma; AGEs, advanced glycation end-products; HIV, human immunodeficiency virus; ATP, adenosine triphosphate; IL-1β, interleukin-1 beta; NPCs, neural progenitor cells; HCF, high fat/cholesterol*.

**Table 2:** **Regulation of connexin- and pannexin-based channels in microglia**.

Effector	Protein involved	Effect on gap junction channel	Effect on hemichannel/pannexon	Technique	Reference
LPS	Cx32	NT	↑	Glutamate release	Takeuchi et al. (2006, 2008)
TNF-α	Cx32	NT	↑	Glutamate release	Takeuchi et al. (2006)
MeCP2 deficiency	Cx32	NT	↑	Glutamate release	Maezawa and Jin (2010)
Amyloid β-peptide	Cx43	NT	↑	Ethidium uptake/patch clamp	Orellana et al. (2011b)
Brain stab wound	Cx43	↑	NT	Lucifer yellow coupling	Eugenín et al. (2001)
Brain stab wound	NF	NF	NT	Sulforhodamine 101 coupling/biocytin coupling	Richter et al. (2014)
Brain stab wound	NF	NF	NT	Sulforhodamine 101 coupling/sulforhodamine B coupling	Wasseff and Scherer (2014)
Calcium ionophore	Cx43?	↑	NT	Lucifer yellow coupling	Martínez et al. (2002)
Calcium chelator	Cx43?	↓	NT	Lucifer yellow coupling	Sáez et al. (2013)
cAMP	Cx43?	NE	NT	Lucifer yellow coupling	Martínez et al. (2002)
CD38 downregulation	Cx43	NT	↑	ATP release/Lucifer yellow uptake	Ma et al. (2014)
cGMP	Cx43?	NE	NT	Lucifer yellow coupling	Martínez et al. (2002)
Cx43^(E2)^	Cx43?	↓	NT	Lucifer yellow coupling	Sáez et al. (2013)
Glioma	NF	NF	NT	Sulforhodamine 101 coupling/biocytin coupling	Richter et al. (2014)
IL-6	Cx43?	↓	↓	Lucifer yellow coupling, ethidium uptake	Sáez et al. (2013)
Lanthanum	Cx43?	↓	↓	Lucifer yellow coupling, ethidium uptake	Sáez et al. (2013)
LPS injection (i.p.)	NF	NF	NT	Sulforhodamine 101 coupling/sulforhodamine B coupling	Wasseff and Scherer (2014)
oATP	Cx43?	↓	NT	Lucifer yellow coupling	Sáez et al. (2013)
Peptidoglycan	Cx43?	↑	NT	Lucifer yellow coupling	Garg et al. (2005)
PMA	Cx43?	↓	NT	Lucifer yellow coupling	Martínez et al. (2002)
Prenatal nicotine and postnatal HFC diet	Cx43	NT	NE	Ethidium uptake	Orellana et al. (2014)
Restraint stress	Cx43	NT	NE	Ethidium uptake	Orellana et al. (2015)
RyR blocker	Cx43	NT	↑	ATP release/Lucifer yellow uptake	Ma et al. (2014)
TNF-α	Cx43?	↑	↑	Lucifer yellow coupling, propidium uptake	Shaikh et al. (2012)
TNF-α + ATP	Cx43?	↑	NT	Lucifer yellow coupling	Sáez et al. (2013)
TNF-α + IL-1β	Cx43?	↑	NT	Lucifer yellow coupling	Sáez et al. (2013)
TNF-α + IFN-γ	Cx43?	↑	↑	Lucifer yellow coupling, ethidium uptake	Sáez et al. (2013)
TNF-α + IFN-γ + ATP	Cx43?	↑	NT	Lucifer yellow coupling	Sáez et al. (2013)
^10^Panx1	Cx43?	↓	NT	Lucifer yellow coupling	Sáez et al. (2013)
Amyloid β-peptide	Panx1	NT	↑	Ethidium uptake/patch clamp	Orellana et al. (2011b)
ATP	Panx1?	NT	↑	Ethidium uptake/yopro uptake	Bernier et al. (2012) and Sáez et al. (2013)
LPS	Panx1	NT	↑	Ethidium uptake/ATP release	Orellana et al. (2013b)
Prenatal nicotine and postnatal HFC diet	Panx1	NT	↑	Ethidium uptake	Orellana et al. (2014)
Restraint stress	Panx1	NT	↑	Ethidium uptake	Orellana et al. (2015)
TGF-β	Panx1	NT	↓	Ethidium uptake	Orellana et al. (2013b)
Zinc	Panx1?	NT	↑	ATP release	Higashi et al. (2011)

*NE, no effect; NT, not tested; NF, not found; LPS, lipopolysaccharide; Mecp2, methyl-CpG binding protein 2; TNF-α, tumor necrosis factor alpha; cAMP, cyclic adenosine monophosphate; cGMP, cyclic guanosine monophosphate; IL-6, interleukin-6; oATP, oxidized adenosine triphosphate; PMA, phorbol-12-myristate-13-acetate; RyR, ryanodine receptor; IFN-γ, interferon gamma; ATP, adenosine triphosphate; IL-1β, interleukin-1 beta; HCF, high fat/cholesterol; TGF-β, transforming growth factor beta*.

## Gap Junction Coupling and its Implications for Microglial Functions

**Figure 2:**
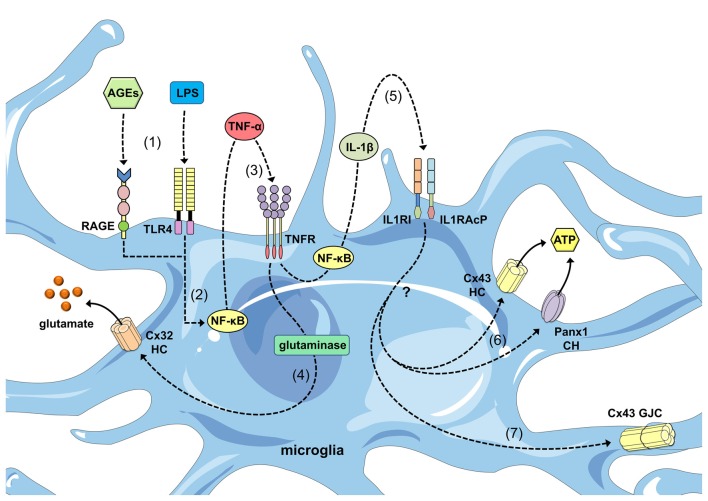
**Regulation of connexin- and pannexin-based channels by cytokines released from activated microglia.** Advanced glycation end-products (AGEs) and lipopolysaccharide (LPS) stimulate the receptor for age (RAGE) and Toll-like receptor 4 (TLR4) receptors (1), respectively, resulting in activation of the nuclear factor-κβ; (NF-κβ) pathway (2). This pathway induces the autocrine/paracrine release of tumor necrosis factor-α; (TNF-α), which acts upon its receptor TNFR1 (3) and leads to the activation of glutaminase and the further release of glutamate through Cx32 HCs (4). In parallel, NF-κβ signaling promotes the autocrine/paracrine release of interleukin-1β; (IL-1β), which acts upon its receptor as well as accessory proteins (IL1RI and IL1RAcP; 5) and leads to the release of ATP through Cx43 HCs and Panx1 channels (CHs) via an unknown mechanism (6). Additionally, IL-1β signaling favors the opening of GJCs composed of Cx43.

Connexin expression among microglia depends heavily on the context of their activation. Although connexin36 (Cx36) and connexin32 (Cx32) are highly expressed in the resting surveillance state (Parenti et al., 2002; Dobrenis et al., 2005; Maezawa and Jin, 2010), Cx43 protein and messenger ribonucleic acid (mRNA) expression is rarely detected under these conditions (Eugenín et al., 2001; Martínez et al., 2002; Rouach et al., 2002; Dobrenis et al., 2005; Garg et al., 2005; Hinkerohe et al., 2005, 2010; Lee et al., 2005; Même et al., 2006; Shaikh et al., 2012; Richter et al., 2014). When microglia shift from resting to activated states in different pathological scenarios, connexin expression increases, particularly connexin29 (Cx29; Moon et al., 2010), Cx32 (Takeuchi et al., 2006; Maezawa and Jin, 2010; Moon et al., 2010), Cx36 (Cepeda et al., 2015) and Cx43 (Eugenín et al., 2001; Garg et al., 2005; Orellana et al., 2011b; Eugenin et al., 2012; Sáez et al., 2013; Talaverón et al., 2014; Table 1). In the first study to demonstrate this phenomenon, reported by Eugenín et al. (2001), stab wound injury or treatment with tumor necrosis factor alpha (TNF-α) plus interferon gamma (IFN-γ) promoted microglial activation and strongly increased Cx43 expression based on immunofluorescence and western blotting analyses. Remarkably, TNF-α plus IFN-γ enabled microglial communication via gap junctions based on dye coupling with Lucifer yellow (LY). This phenomenon occurs in a Cx43-dependent manner because microglia cultured from Cx43 knock-out (KO) mice did not exhibit GJC up-regulation following treatment with the same mixture of pro-inflammatory agents (Eugenín et al., 2001). Similar findings involving advanced glycation end-products (AGEs) and TNF-α were reported by Shaikh et al. (2012) AGE treatment increased Cx43 expression and the release of TNF-α in human microglial CHME-5 cells via a mechanism dependent on activation of the receptor for AGE (RAGE). Importantly, treatment with TNF-α for 6 h mimicked AGE-induced Cx43 expression, but also evoked the development of direct microglia-to-microglia coupling and HC opening. Therefore, the AGE-induced release of TNF-α by microglia exerted autocrine/paracrine enhanced effects on both GJC and HC functions (Shaikh et al., 2012; Table 2; Figure 2).

Microglia are rapidly activated in response to tissue damage (Davalos et al., 2005), which initiates the complex production of cytokines and gliotransmitters (e.g., ATP/ADP) that act in an autocrine/paracrine manner to specifically govern dynamic changes in the microglial phenotype (Chakfe et al., 2002; Inoue, 2002; Seo et al., 2008). Thus, these modulators are not simple on/off switches that control microglial activation but instead demonstrate synergism, cooperation and even antagonism. This phenomenon is evident in the induction of microglial coupling triggered by TNF-α, as previously mentioned (Figure 2). In a study by Sáez et al. (2013), ATP, IFN-γ or interleukin-6 (IL-6) accelerated, delayed or prevented TNF-α-evoked LY coupling in cultured microglia, respectively (Figure 1). Because oxidized ATP (P2X receptor blocker), 1,2-bis(o-aminophenoxy)ethane-N,N,N′,N′-tetraacetic acid, BAPTA, Ca^2+^ chelator) and IL-1ra (IL-1β receptor antagonist) prevented microglial coupling caused by TNF-α plus ATP, increases in the intracellular free Ca^2+^ concentration ([Ca^2+^]_i_) induced via the activation of P2X receptors led to the autocrine release of IL-1β and further upregulation of GJC functions (Sáez et al., 2013; Figure 2). Consistent with this line of thought, P2X receptor activation increases [Ca^2+^]_i_ in microglia (Ferrari et al., 1996) and promotes gap junction communication (Martínez et al., 2002). In seminal studies performed by Martínez et al. (2002), LY coupling evoked by the Ca^2+^ ionophore 4Br-A23187 correlated with elevated Cx43 protein and mRNA levels. Although the increase in [Ca^2+^]_i_ is a conserved mechanism to directly inhibit gap junctions formed by several connexins (Peracchia, 2004), it is unknown whether this phenomenon is unique to microglia or the indirect result of downstream effectors of Ca^2+^ that regulate the sorting, synthesis and/or degradation of Cx43 GJCs.

The previously described microglial coupling not only begins during cytokine treatment but is also initiated when microglia are exposed to bacteria-derived agents (Table 2). In studies in primary microglia performed a decade ago, *Staphylococcus aureus*-derived peptidoglycan (PGN) increased Cx43 mRNA and protein levels as measured by reverse transcription polymerase chain reaction (RT-PCR), western blotting and immunofluorescence analysis (Garg et al., 2005). Notably, Cx43 upregulation was accompanied by the gap junction-dependent spread of LY among microglia, and this response was completely blunted by 18-α-glycyrrhetinic acid, which is a well-known gap junction blocker (Garg et al., 2005). Microglial coupling triggered by PGN relies on the direct action of this molecule on pattern recognition receptors, including CD14 and Toll-like receptor 2 (TLR2). Supporting this notion, the direct activation of TLR2 upregulates Cx43 mRNA expression and functional communication through GJCs in intestinal epithelial cells (Ey et al., 2009). Alternatively, nuclear factor κB (NF-κB) and mitogen-activated protein kinase (MAPK) signaling promote the autocrine/paracrine release of cytokines and inflammatory mediators following PGN-induced TLR2 activation (Akira et al., 2001), particularly TNF-α and IL-1β, which are well known inducers of gap junction functions in microglia (Eugenín et al., 2001; Shaikh et al., 2012; Sáez et al., 2013).

Does functional gap junctional communication occur between microglia and other brain cells? According to Dobrenis et al. (2005) microglial cells possess the potential not only to build functional gap junctions through Cx36 but also to establish this type of communication with hippocampal neurons. These authors observed Cx36 expression in microglia and the presence of electrical coupling between microglia with low unitary conductance (<20 pS) and very low voltage sensitivity, which are properties of Cx36 GJCs but not other connexins (Srinivas et al., 1999; Teubner et al., 2000). These heterocellular interactions mediated by neuro-microglial gap junctions demonstrate anatomical and functional correlations neural progenitor cells (NPCs) isolated from the subventricular zone (SVZ). Talaverón et al. (2014) identified gap junctions between implanted NPCs and host microglia at the ultrastructural level in animals lesioned by axotomy. In a follow-up study, NPCs and microglia established functional gap junctions during co-culture as measured by LY dye transfer (Talaverón et al., 2015). Multiple connexins are likely involved in this phenomenon because both Cx45 and Cx43 are expressed in NPCs (Talaverón et al., 2015), and both connexins have also been found in microglia (Dobrenis et al., 2005; Higashi et al., 2011). Thus, coupling between microglial cells and NPCs sustains the reciprocal signaling necessary for the proper development of neurogenic niches, including the SVZ (Talaverón et al., 2015). Consistent with this view, microglia and NPCs exhibit close spatial interactions in the SVZ neurogenic niche, and their interplay affects microglial functions and NPC fate (e.g., proliferation, differentiation and survival; Mäkelä et al., 2010; Mosher et al., 2012; Liu et al., 2013; Shigemoto-Mogami et al., 2014). Alternatively, given that the conservation of implanted NPCs depends on the immunomodulatory and neuroprotective molecules released by microglia (Martino and Pluchino, 2006), gap junction coupling among these cell types has the potential to be critical for experimental neural cell therapies that exert beneficial effects on recovery after brain injury.

Recently, two independent groups questioned the existence of functional microglial coupling *ex vivo*. Employing brain slices derived from CX3CR1-EGFP mice, Wasseff and Scherer (2014) failed to detect the transfer of sulforhodamine-B (SR-B) between injected microglia and any other cell type, including EGFP-positive microglia. Similar observations were also reported for neocortex slices from an Alzheimer’s mouse model and mice exposed to a single lipopolysaccharide (LPS) injection (Wasseff and Scherer, 2014). Furthermore, control microglia or microglia found within the glioma environment and stab wound areas were not coupled by gap junctions based on the transfer of biocytin or sulforhodamine101 (SR101; Richter et al., 2014). Considering this controversy in light of early experiments favoring *in vitro* microglial coupling (Eugenín et al., 2001; Martínez et al., 2002; Garg et al., 2005), one may question whether SR-B, biocytin or SR101 are appropriate dyes to observe coupling among microglia. GJCs formed by different connexins exhibit specific permeability properties depending on the size, shape and charge of the molecules (Harris, 2007). Most studies claiming functional coupling among microglia were performed by microinjecting LY (Eugenín et al., 2001; Martínez et al., 2002; Garg et al., 2005; Shaikh et al., 2012; Sáez et al., 2013). Therefore, the discrepancies surrounding functional coupling likely arise from the different methods employed to assess functional gap junction communication.

Activated microglia are the major antigen presenting cells (APCs) in the CNS (Almolda et al., 2015). APCs take up antigens and process them into proteolytic peptides to generate major histocompatibility complexes (MHCs), which are located on the cell surface and initiate a proper adaptive immune response by activating CD4^+^ and CD8^+^ T cells (van Kasteren et al., 2014). APC-induced T cell stimulation relies on a mechanism involving the assembly of a specialized supramolecular structure known as the immunological synapse. This structure engages T cell receptors (TCRs) through MHC-peptide complexes on APCs (Grakoui et al., 1999). In the pioneering work by Neijssen et al. (2005), the transfer of small peptides (~1.8 kDa) between Cx43-transfected cells represents a new mechanism for antigen cross-presentation between APCs and T cells. In this context, dendritic cells (DCs), which are professional APCs, establish functional GJCs among themselves and with T cells, thereby contributing to antigen transfer and DC activation as well as cross-presentation during T cell activation at the immunological synapse (Matsue et al., 2006; Corvalán et al., 2007; Mendoza-Naranjo et al., 2007, 2011; Elgueta et al., 2009). Whether gap junctions are crucial for the hypothetical transfer of antigen peptides among activated microglia or whether these channels cross-present antigens to T cells remains an open question. Microglia possess the majority of the machinery to cross-present antigens. Indeed, microglia express MHCs and co-stimulatory molecules following a wide range of brain injuries, including LPS-induced inflammation, ischemia, axotomy and experimental autoimmune encephalomyelitis (Almolda et al., 2015).

## Functional Hemichannels and Pannexons in Microglia

To date, the functional expression of HCs in microglia has been documented in different studies (Table 2). Pioneering observations by Takeuchi et al. (2006) noted that Cx32 possesses the ability to form functional HCs in microglia. TNF-α induces microglial glutamate release through the opening of Cx32 HCs, a response dramatically suppressed by a mimetic peptide against Cx32 (^32^Gap27; Takeuchi et al., 2006; Figure 2). Moreover, glutamate release strongly correlates with the number of Cx32 HCs available on the microglial cell surface, suggesting a possible role for TNF-α in the sorting and/or trafficking of Cx32. The TNF-α-induced release of glutamate via Cx32 HCs triggers neuritic beading and neuronal death, as observed by phase contrast microscopy and terminal deoxynucleotidyl transferase-dUTP nick end labeling (TUNEL) analysis (Takeuchi et al., 2006). Thus, the opening of glial cell HCs is detrimental for normal neuronal functions. These findings were corroborated by other groups who performed dye uptake experiments in which TNF-α by itself induced HC opening in human microglial CHME-5 cells (Shaikh et al., 2012). Similarly, TNF-α plus IFN-γ evoked dye uptake in EOC20 microglial cells via a mechanism sensitive to the pharmacological inhibition of HCs and pannexons (Sáez et al., 2013). These findings, as well as the observation that TNF-α plus IFN-γ increases the expression of Cx43 and Panx1, favors a role for Cx43 HCs and Panx1 channels as major targets of inflammatory mediators (Sáez et al., 2013).

Reinforcing the hypothesis that microglial Cx32 HCs are key players in neurotoxicity, glutamate released via these protein conduits was critical for the induction of neuronal damage during brain ischemia (Takeuchi et al., 2008) and experimental autoimmune encephalomyelitis (Shijie et al., 2009) in studies from Suzumura’s group. Unfortunately, the involvement of Cx32 HCs in these brain pathologies was deduced based on the positive outcome of disease progression achieved by carbenoxolone (CBX) administration (Takeuchi et al., 2008; Shijie et al., 2009). CBX is a widely used gap junction blocker that also inhibits Panx1 channels (~5 μM) but does not distinguish between Panx1 channels and HCs at concentrations above 50 μM (Bruzzone et al., 2005; D’hondt et al., 2009). Therefore, it is premature to draw conclusions regarding the true participation of microglial Cx32 HCs in the progression of brain disorders in the studies described above. However, a recent study substantiated the involvement of microglial Cx32 HCs in Rett syndrome, which is a neurodevelopmental disorder that affects young girls and is primarily caused by loss-of-function mutations in the X-linked MECP2 encoding methyl-CpG-binding protein 2 (MeCP2; Chahrour and Zoghbi, 2007). In this study, MeCP2-null microglia released large amounts of glutamate due to increased production by glutaminase and increased release through Cx32 HCs (Maezawa and Jin, 2010). Strikingly, the Cx32 HC blockers ^32^Gap27 and ^32^Gap24 dramatically prevented microglia-mediated dendritic atrophy and the reduction of dendritic (MAP2 and Ac-TN) and postsynaptic protein density (PSD95 and GRIP1) and the glutamate receptor subunits NR1, GluR2/3, and GluR6/7 (Maezawa and Jin, 2010). Thus, Cx32 HCs represent potential pharmacological targets for therapies to counter the harmful effects linked to microglial dysfunction and miscommunication with neurons during Rett syndrome.

Microglia under a resting surveillance state express significant levels of Panx1 (Higashi et al., 2011; Orellana et al., 2011b, 2014; Rigato et al., 2012; Sáez et al., 2013) but not Panx2 (Rigato et al., 2012). Similar to the observations with connexins, Panx1 is upregulated in microglia stimulated with different pro-inflammatory agents, including IFN-γ (Shestopalov and Slepak, 2014), amyloid-β peptide (Aβ; Orellana et al., 2011b), TNF-α plus ATP (Sáez et al., 2013), TNF-α/IFN-γ (Sáez et al., 2013) and TNF-α/IL-1β (Sáez et al., 2013). The functional expression of pannexons in microglia is a relatively new discovery stemming from studies in which Aβ induces dye uptake and macroscopic currents in these cells (Orellana et al., 2011b). These responses are inhibited by mimetic peptides and pharmacological blockers against Panx1 channels (^10^panx1, E1b and probenecid) or Cx43 HCs (Gap26, Gap27 and Cx43^E2^), whereas microglial cultures from Cx43-null mice are unaffected by Aβ treatment (Orellana et al., 2011b). The currents recorded at negative holding potentials (−60 mV) reveal unitary events with conductances near ~500 and ~220 pS; these conductances do not differ greatly from those observed for Panx1 channels and Cx43 HCs (Contreras et al., 2003; Bao et al., 2004). Additionally, channel opening correlates with microglial activation, increased surface levels of Panx1 and Cx43, and the prominent release of glutamate and ATP via a pathway sensitive to Panx1 channel and Cx43 HC blockers (Orellana et al., 2011b). Notably, N-methyl-D-aspartate (NMDA) and P2X_7_ receptor activation triggered by these gliotransmitters results in the opening of Panx1 channels in neurons, resulting in detrimental consequences for neuronal survival (Orellana et al., 2011b).

The question of whether microglial pannexons and/or HCs indeed participate in the onset and progression of Alzheimer’s disease (AD) remains unanswered. A recent study by Takeuchi et al. (2011) described a possible role for microglial Cx32 HCs as major regulators of the brain damage observed in AD. The BBB-permeable Cx32 HC blocker INI-0602 not only ameliorated symptoms in two amyotrophic lateral sclerosis (ALS) mouse models, but also improved memory deficits in the APP/PS1 transgenic model of AD (Takeuchi et al., 2011). Interestingly, INI-0602 successfully inhibited microglial glutamate release *in vivo*, potentially implicating microglial HC-dependent excitotoxicity in disease progression in ALS and AD mouse models. Although INI-0602 reduces Cx32 HC-mediated glutamate release in cultured microglia (Takeuchi et al., 2011), it will be interesting to examine whether this HC blocker maintains its specificity over other channels *in vivo*. The functional detection of microglial HCs and/or pannexons in brain slices derived from AD mouse models using pharmacological (e.g., mimetic peptides) and molecular (e.g., protein downregulation) approaches will fully elucidate the involvement of these microglial channels in AD.

Recently, the opening of microglial pannexons was described in mouse models of stress-related mental disorders, such as anxiety and major depression. In dye uptake experiments, acute and chronic restraint stress protocols activated microglial Panx1 channels in hippocampal slices, with chronic restraint stress exerting the strongest effects on these channels (Orellana et al., 2015). This response did not occur in hippocampal slices from Panx1 null mice and was fully suppressed by NMDA and P2X_7_ receptor blocking (Orellana et al., 2015). Restraint stress also elicits the opening of astroglial and neuronal HCs and the concomitant involvement of the Cx43 and Panx1 channel-forming proteins in these responses, respectively (Orellana et al., 2015). Interestingly, glutamate and ATP release is highly promoted in hippocampal slices derived from mice subjected to restraint stress, but this phenomenon is entirely blunted after treatment with Panx1 channel, Cx43 HC and NMDA and P2X_7_ receptor inhibitors (Orellana et al., 2015). The authors proposed that chronic restraint stress might increase brain levels of glucocorticoids (GCs), resulting in further activation of the NMDA/P2X_7_ receptors in microglia and astrocytes. Consistent with this idea, chronic stress triggers NMDA and GG receptor-mediated microglial activation (Nair and Bonneau, 2006), whereas GC exposure primes cytokine release from microglia *ex vivo* (Frank et al., 2007); the latter effect is a well-known inducer of ATP and glutamate release through glial cell HCs (Takeuchi et al., 2006; Avendaño et al., 2015). In summary, HC opening alters brain concentrations of different transmitters and thus represents an overlooked form of miscommunication that modifies the proper function of neuronal circuits in people suffering from stress-related mental disorders.

## Crosstalk Between Astrocytes and Microglia Regulates Connexin and Pannexin-Based Channels

Microglia and astrocytes respond to brain injury with distinctive spatial and temporal activation patterns and their crosstalk appears to be critical for neuronal outcomes (Liu et al., 2011). The release of gliotransmitters, cytokines and growth factors represents the primary mechanism by which glial cells communicate with one another and respond to neural activity (Färber and Kettenmann, 2006; Koizumi, 2010). Activated microglia modulate connexin expression and functional GJC and HC activity in astrocytes (Rouach et al., 2002; Faustmann et al., 2003; Hinkerohe et al., 2005; Même et al., 2006; Retamal et al., 2007a; Orellana et al., 2011a). Normally, astrocytes are strongly coupled through gap junctions and express high levels of Cx43 (Giaume et al., 1991). However, when they are cultured with resting microglia, Cx43 levels and dye coupling are obviously reduced (Rouach et al., 2002) and potentiated by LPS (Même et al., 2006). Interestingly, the above response is mimicked with conditioned media (CM) harvested from microglia pre-treated with LPS, implying soluble factors released by microglia modulate astrocytes (Même et al., 2006). Indeed, studies involving Enzyme-Linked ImmunoSorbent Assay (ELISA), immunoneutralization and cytokine receptor blocking reported that IL-1β and TNF-α are soluble factors affecting Cx43 expression and GJC functions in astrocytes (Même et al., 2006). Importantly, the influence of microglia on the function of astroglial connexins extends beyond gap junctions and also impacts the opening of HCs. Astrocytes exposed to CM from LPS-stimulated microglia or IL-1β plus TNF-α exhibit a conspicuous increase in HC activity as determined from unitary current conductance measurements and dye uptake experiments (Retamal et al., 2007a). This effect occurs in parallel with the inhibition of dye coupling; thus, both cytokines released by microglia regulate gap junctions and HCs in an opposing manner in astrocytes (Retamal et al., 2007a). Notably, p38 MAPK pathway inhibition completely reduces the dual influence of microglia in astroglial connexin channels (Retamal et al., 2007a). Supporting these findings, WIN and methanandamide, which are two well-known cannabinoids that counteract p38 MAPK signaling (Carracedo et al., 2004), impede the LPS-mediated release of IL-1β and TNF-α from microglia, thereby limiting their control of astroglial connexins (Froger et al., 2009).

**Figure 3:**
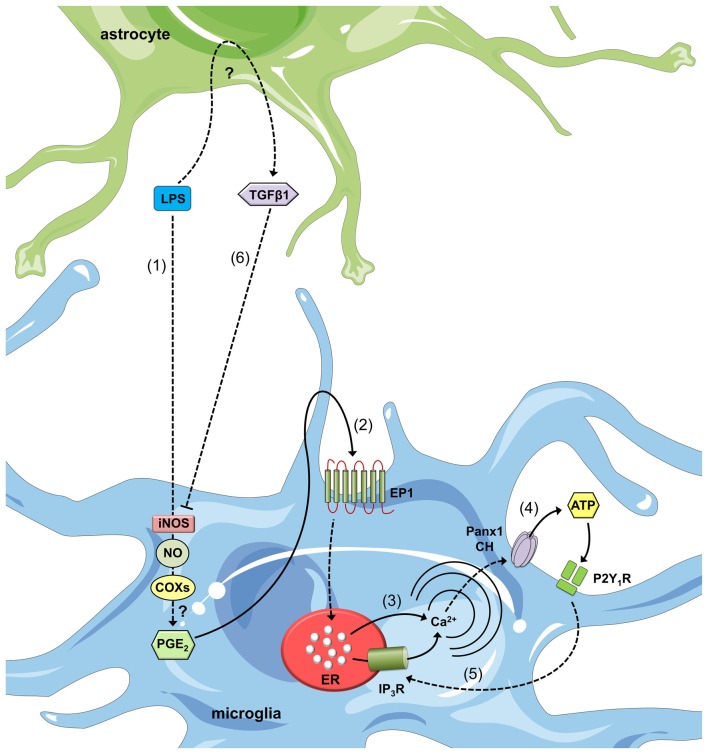
**Astroglial modulation of ATP-induced Ca^2+^ dynamics in LPS-stimulated microglia.** After LPS stimulation, microglia respond with intracellular signal transduction, leading to iNOS activation, nitric oxide (NO) production, cyclooxygenase (COX) activation and prostaglandin E2 (PGE_2_) production via unknown mechanisms (1). PGE_2_ released by microglia binds to the EP1 metabotropic receptor (2) to induce Ca^2+^ release from intracellular stores (3). This release increases [Ca^2+^]_i_, which is known to open Panx1 channels (CHs) and subsequently release ATP (4). ATP released via Panx1 CHs and its degradation to ADP activate P2Y_1_ receptors, which induce IP_3_ receptor activation and the further release of Ca^2+^ stored in the endoplasmic reticulum (5). Astrocytes stimulated with LPS release transforming growth factor β1 (TGFβ1) (6), which inhibits LPS-induced intracellular signal transduction and causes iNOS activation. An alternative negative feedback loop mediates the inhibitory effects of ATP on Panx1 channels (not depicted). Finally, paracrine release of ATP from microglia acts upon neighboring or distant microglia, resulting in an additional feed-forward mechanism (not depicted).

Recently, factors released by astrocytes were shown to counteract the immunomodulatory actions of LPS in microglia, including ATP release (Orellana et al., 2013b). In a recent study, LPS-stimulated microglia produced large amounts of nitric oxide (NO), which was accompanied by a significant increase in [Ca^2+^]_i_ and ATP release (Orellana et al., 2013b; Figure 3). These responses were effectively blocked by L-N6, NS-398, SC-560 and SC 19220, which are selective inhibitors of iNOS, cyclooxygenase-1 (COX_1_), cyclooxygenase-2 (COX_2_) and the PGE_2_ receptor EP_1_, respectively (Orellana et al., 2013b). Interestingly, LPS-induced ATP release was not detected after blocking Panx1 channels with probenecid and ^10^panx1 or in microglia treated with siRNA targeting Panx1 (Orellana et al., 2013b). This peculiar pharmacology suggested a direct link between LPS and Panx1 channel opening, which occurred in response to NO production, COX activation and PGE_2_ release_._ Remarkably, Transforming growth factor beta 1 (TGF-β1) release by activated astrocytes completely abolished the LPS-induced increase in NO production, [Ca^2+^]_i_, ATP release and Panx1-dependent dye uptake (Orellana et al., 2013b; Figure 3). Moreover, the Panx1-dependent release of ATP and subsequent activation of the P2Y_1_ and P2X_7_ receptors were crucial for the preservation of LPS-mediated NO production in microglia (Orellana et al., 2013b). Subsequent experiments corroborated the connection between iNOS/COXs/EP_1_ receptor signaling and the functional opening of Panx1 channels in microglia. Prenatal nicotine exposure and a postnatal high-fat/cholesterol diet after weaning increase the opening of astroglial Cx43 HCs and Panx1 channels in microglia and neurons (Orellana et al., 2014). The opening of microglial pannexons is not paralleled by significant changes in Panx1 expression but is associated with the release of ATP via a mechanism dependent on the activation of the iNOS/COX_2_/EP_1_ receptor pathway and signaling via P2X_7_ receptors (Orellana et al., 2014).

How does the ATP released through microglial Panx1 channels contribute to cell-to-cell communication in the brain parenchyma? ATP is a crucial transmitter involved in the crosstalk between brain cells (Fields and Burnstock, 2006) and has often been linked with the activation and chemotaxis-related features of microglia (Honda et al., 2001). In the CNS, one of the major signs driving microglia into an “activated” state is a prominent rise in extracellular ATP levels, potentially in response to tissue injury. Given this background, microglial sensitivity to ATP relies on the expression of the two purinergic receptor families: G-protein coupled-type P2 receptors (P2Y receptors, P2YRs) and ionotropic P2 receptors (P2X receptors, P2XRs; Inoue, 2002). Upon their activation, P2YRs/P2XRs induce membrane ruffling and ramification of cultured microglia (Nolte et al., 1996) as well as the release of different messengers and signaling molecules, such as TNF-α and IL-1β (Inoue, 2002). The majority of these paracrine mediators reinforce microglial activation (Chao et al., 1995; Kuno et al., 2005) and lead to the opening of microglial pannexons and HCs (Takeuchi et al., 2006; Shaikh et al., 2012; Sáez et al., 2013). In leading-edge studies by Davalos et al. (2005), extracellular ATP controlled microglial chemotactic responses in the brain parenchyma; moreover, its release from injured tissue (particularly astrocytes) mediated a rapid microglial reaction towards the damage site. Because CBX and flufenamic acid (nonspecific blockers of HCs and pannexons) strongly inhibit microglial responses towards a site of injury, both channels were suggested to be major astroglial conduits for ATP release during rapid microglial responses to local brain trauma (Davalos et al., 2005). Although a great deal remains to be learned about the events affecting the opening of HCs and/or pannexons in microglia during brain trauma, this study demonstrated a role for connexins and pannexins in microglial branch dynamics.

Astrocytes are the primary partnering cells that share ATP-mediated communication with microglia. Astrocytes release ATP through Cx43 HCs and/or Panx1 channels (Cotrina et al., 1998; Braet et al., 2003; Iglesias et al., 2009; Garré et al., 2010; Orellana et al., 2011a,b; Suadicani et al., 2012), resulting in the activation of P2XRs/P2YRs and the regenerative release of ATP via these channels (Iglesias et al., 2009; Garré et al., 2010; Suadicani et al., 2012). This mechanism of ATP-induced ATP release is critical for the spread of Ca^2+^ waves (Guthrie et al., 1999; Anderson et al., 2004) and constitutes a critical pathway through which astrocytes exert their immunomodulatory actions over microglia (Verderio and Matteoli, 2001; Schipke et al., 2002). Indeed, purinergic receptors and the activation of HCs and pannexons represent an essential mechanism for the maintenance of the regenerative release of ATP in microglia because acute application of this gliotransmitter induces the opening of Cx43 HCs and Panx1 channels in these cells (Bernier et al., 2012; Sáez et al., 2013). How does ATP activate P2XRs in microglia? Extracellular ATP produces different outcomes in terms of the P2XR-mediated activation of macrophages and microglia depending on the treatment period and concentration used (Baroja-Mazo et al., 2013). For P2X_7_Rs, brief treatment with ATP stimulates small cationic currents of 10–30 pS, whereas repetitive or long-lasting application of ATP generates large currents of ~440 pS and the rapid uptake of large molecules (up to 900 kDa). Potentially, P2X_7_R conductance increases over time, resulting in the formation of a large pore to facilitate the passage of molecules; alternatively, P2X_7_Rs activates a second non-selective permeability pathway (Baroja-Mazo et al., 2013). Panx1 channels mediate P2X_7_R-dependent permeability for large molecules in macrophages (Pelegrin and Surprenant, 2006) and microglia (Bernier et al., 2012) but do not participate in microglial P2X_4_R pore formation (Bernier et al., 2012). Coincident findings related to the latter observation were recently described in mouse peritoneal macrophages (Seil et al., 2010), and a Panx1-independent model has been proposed for P2X_2_R large molecule permeability (Chaumont and Khakh, 2008). Future studies will elucidate the participation of Panx1 channels in the long-lasting activation of other P2XRs in addition to P2X_7_Rs in microglia.

P2X_7_R activation drives the release of ATP in microglia via at least one mechanism involving HCs and/or pannexons. Although P2X_7_R activation increases [Ca^2+^]_i_ (Baroja-Mazo et al., 2013) and opens Cx43 HCs and Panx1 channels (Locovei et al., 2006b; De Vuyst et al., 2009; De Bock et al., 2012; Wang et al., 2012), the Panx1-dependent release of ATP relies on protein-protein interactions between P2X_7_Rs and Panx1 (Locovei et al., 2007). Supporting this idea, Panx1 co-immunoprecipitates with P2X_7_Rs (Pelegrin and Surprenant, 2006; Silverman et al., 2009; Li et al., 2011; Poornima et al., 2012; Hung et al., 2013), and proline 451 in the C-terminal tails of these receptors has been implicated in this interaction (Iglesias et al., 2008; Sorge et al., 2012). P2YRs activation constitutes a second mechanism by which ATP is released from microglia. Because P2YRs mediate Ca^2+^ release from intracellular stores, they induce HC/pannexon opening linked to the consequential release of ATP from microglia, which was previously demonstrated for other cell types (Locovei et al., 2006a; Orellana et al., 2012a; Zhang et al., 2012). Although some observations support exocytosis as the predominant route for ATP release in microglia (Imura et al., 2013), Panx1 channels and Cx43 HCs facilitate its release after stimulation with LPS (Orellana et al., 2013b), Aβ (Orellana et al., 2013b) or ZnCl_2_ (Higashi et al., 2011).

Interestingly, P2X_7_R-dependent opening of Panx1 channels has been linked to the secretion of IL-1β by a mechanism involving the activation of the inflammasome (Pelegrin and Surprenant, 2006, 2007, 2009; Kanneganti et al., 2007). During an infection, innate immune cells recognize different pathogen-associated molecular patterns (PAMPs) and damage-associated molecular patterns (DAMPs) expressed and released by invading pathogens and dying cells, respectively (Bianchi, 2007). Members of the NOD-like receptor (NLR) family recognize PAMPs and DAMPs and in association with the adaptor apoptosis speck-like protein containing a CARD (ASC) constitute the inflammasome platform (Martinon et al., 2002), which is critical for caspase-1 activation, production of the IL-1β and proper host defense response (Netea et al., 2010). ATP is a well-known DAMP released from damaged or dying cells after trauma that promotes inflammasome activation by acting at P2X_7_R (Ayna et al., 2012). Protein-to-protein interaction between P2X_7_R and Panx1, as well as increase in extracellular K^+^ stimulate Panx1 channel opening, thus releasing more ATP into the extracellular space (Pelegrin and Surprenant, 2006, 2007, 2009; Kanneganti et al., 2007). In neurons and astrocytes, opening of Panx1 channels leads to caspase-1 activation by a mechanism involving the association of Panx1 with components of the multiprotein inflammasome complex, including the P2X_7_R (Silverman et al., 2009; Murphy et al., 2012; Minkiewicz et al., 2013). Although IL-1β secretion in microglia depends on activation of the inflammasome (Terada et al., 2010; Burm et al., 2015), whether Panx1 channels participate in this process remain to be elucidated.

In seminal studies by Bruzzone et al. (2001), Cx43 HCs promoted cyclic ADP-ribose (cADPR) signaling by inducing the release of NAD^+^. After cyclization of NAD^+^ by CD38, this ectoenzyme produces cADPR, which is a potent endogenous agonist of ryanodine receptors (RyRs) that serves as a universal mobilizer of Ca^2+^ from intracellular stores (Malavasi et al., 2008). Moreover, HCs not only mediate the outward passage of NAD^+^ but also import cADPR into macrophages to activate intracellular Ca^2+^ mobilization (Song et al., 2011). Interestingly, CD38 plays a key role in the basal survival of microglia due to its inhibitory effects on Cx43 HC-dependent ATP release (Ma et al., 2014). Downregulation of CD38 or pharmacological inhibition of RyRs increases ATP release, microglial activation and death via a mechanism involving the opening of Cx43 HCs. Molecular (siRNA) and pharmacological inhibition of Cx43 HCs dramatically ameliorates the decreased CD38/cADPR signaling-induced release of ATP, NO production and apoptosis by microglia (Ma et al., 2014). Cx43 HC opening and ATP release are likely caused by the production of superoxide because treatment with the broadly used antioxidant NAC strongly attenuates RyR inhibition-mediated dye uptake in microglia (Ma et al., 2014). Accordingly, oxidative stress opens Cx43 HCs in astrocytes (Retamal et al., 2006) and HeLa Cx43 transfectants (Retamal et al., 2007b). The Cx43 HC-dependent release of ATP activates P2X_7_R, resulting in a mechanism of ATP-induced ATP release that leads to microglial activation and cell death. Although ATP-mediated HC opening has been linked to cell death in neurons and astrocytes (Orellana et al., 2011a,b; Rovegno et al., 2015), additional studies are required to fully elucidate whether similar mechanisms result in microglial cell death.

Together, this evidence supports the hypothesis that regenerative Panx1/Cx43-dependent ATP release from astrocytes and microglia relies on purinergic, CD38/cADPR and Ca^2+^ signaling, resulting in further stimulation of neighboring microglia (Fontainhas et al., 2011; Orellana et al., 2013b; Ma et al., 2014). Thus, P2YR/P2XR activation is turned off in part by ATP diffusion to distal regions as well as the desensitization of these receptors and the degradation of extracellular ATP by exonucleases (Fields and Burnstock, 2006). Similarly, ATP directly inhibits Panx1 channels (Qiu and Dahl, 2009), thereby constituting an alternative negative feedback loop in the glial network. Another regulatory mechanism relies on the inhibitory effects of the increase in [Ca^2+^]_i_ over Cx43 HCs due to purinergic signaling activation. Accordingly, Cx43 HCs exhibit a biphasic and bell-shaped dependency on [Ca^2+^]_i_ (i.e., concentrations below 500 nM result in their activation, whereas concentrations over 500 nM trigger a negative feedback loop; De Vuyst et al., 2009; De Bock et al., 2012; Wang et al., 2012). In the inflamed CNS, astrocytes potentially suppress this self-perpetuating mechanism of ATP release in microglia by secreting anti-inflammatory factors such as TGF-β, as previously observed (Orellana et al., 2013b).

## Pannexons Regulate Microglial Migration and Morphology

Based on a large body of evidence, neurons are an important source of molecules that dictate the morphology, dynamic behavior and chemotaxis of microglia (Ey et al., 2009; Domercq et al., 2013). In particular, pannexons play a crucial role in this crosstalk. According to Fontainhas et al. (2011), the Panx1-dependent release of ATP in response to glutamatergic neurotransmission increased dendritic morphology and process dynamics in microglia in retinal explants. Notably, ATP-induced microglial changes were observed even upon glutamatergic blockade, whereas purinergic receptor inhibition decreased this response despite glutamatergic stimulation, suggesting ATP release occurs “downstream” of glutamate receptor activation (Fontainhas et al., 2011). Although the identity of the cells responsible for ATP release in the retina has not been reported, possible candidates include astrocytes, Müller cells, neurons and microglia themselves.

A recent study that performed *in vivo* time-lapse imaging of both microglial morphology and neuronal activity in the optic tectum of larval zebrafish revealed the crucial role of Panx1 channels on tectal neurons in the dynamic orientation of resting microglial processes (Li et al., 2013). Membrane depolarization evoked large probenecid-sensitive outward currents in tectal neurons but not in microglia. Moreover, blockade of Panx1 channels with probenecid or CBX significantly reduced the number of bulbous endings (microglial processes that dynamically contact the neuronal soma; Li et al., 2013). Acute treatment with the ATP-hydrolyzing enzyme apyrase or the purinergic receptor blocker suramin strongly reduced the percentage of bulbous endings, suggesting the involvement of extracellular ATP and/or ADP in regulating the baseline motility of resting microglial processes (Li et al., 2013). ATP and purinergic signaling have been linked to microglial migration towards distant lesions in leeches (Samuels et al., 2010, 2013). In the leech, a single giant glial cell with structural and functional features similar to mammalian astrocytes and oligodendrocytes ensheathes axon tracts that connect neuronal cell bodies (Deitmer et al., 1999). Apyrase and CBX inhibit the ATP-dependent migration of microglia and their aggregation at lesions (Samuels et al., 2013). The specific downregulation of innexons (i.e., the pannexon homologs in non-chordates) in the giant glia dramatically reduce dye efflux and microglial migration, suggesting the requirement for innexon-dependent release of ATP from glia for microglial chemotaxis at sites of injury (Samuels et al., 2013). Although the question of whether astroglial or neuronal pannexons exert differential influences over microglial migration depending on the context of injury remains open, purinergic signaling is clearly part of this process.

## Conclusions and Future Directions

The functional state of HCs, pannexons and GJCs is finely regulated during inflammation in microglial cells. In this context, different PAMPs (e.g., AGEs, LPS) and DAMPs (e.g., ATP) are recognized by microglial pattern recognition receptors (PRRs), resulting in activation of the NF-κβ pathway and the inflammasome complex. These pathways induce the autocrine/paracrine release of pro-inflammatory cytokines (e.g., IL-1β and TNF-α), which activate the opening of connexin- and pannexin-based channels and the release of ATP through Cx43 HCs and Panx1 channels.

**Figure 4:**
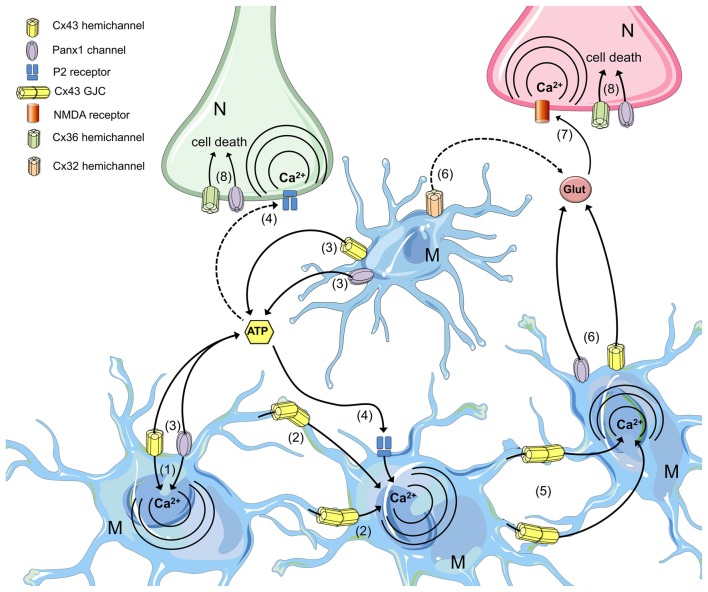
**The roles of microglial connexin- and pannexin-based channels in neurodegeneration.** Under progressive inflammatory conditions, cytokine production and redox imbalance increase the opening of HCs and pannexons in microglia, permitting the influx of Ca^2+^ (1) and its spread to neighboring cells through GJCs (2). Hemichannel and pannexon opening in microglia favor the release of ATP (3), which diffuses through the extracellular space to activate membrane purinergic (P2) receptors (4). Intercellular Ca^2+^ waves propagated via GJCs increase the [Ca^2+^]_i_ in distant microglia (5), facilitating the release of glutamate through microglial HCs/pannexons (6) and the further activation of neuronal n-methyl-d-aspartate (NMDA) receptors (7). P2 and NMDA receptor activation in neurons increase the activity of neuronal Panx1 channels and Cx36 HCs, impairing the electrochemical and Ca^2+^ imbalance that results in cell death (8).

Microglial activation is a highly dynamic and complex process whose development is supported by the transfer of messengers through GJCs and paracrine signaling via the functional opening of HCs and pannexons. The passage of molecules and ions likely relies on the activation of different intracellular pathways based on the degree and stage of microglial activation. Therefore, gap junction communication reflects the ability of an activated microglial cell to recruit resting microglia or to establish direct communication with other cells in the CNS to favor cross-presentation at the immune synapse or promote the repair of damaged tissue (Figure 4). Alternatively, HC/pannexon opening sustains the microglial chemotactic responses and dynamics necessary to ensure proper microglial migration to injury sites. Although the impact of the increased microglial GJC activity on neuronal survival is unknown, the opening of microglial HCs and pannexons certainly lead to neuronal damage (Figure 4). Most studies suggest that the release of gliotransmitters (e.g., ATP, glutamate) through Cx43/Cx32 HCs and Panx1 channels is critical to induce neuronal death by a mechanism involving the activation of NMDARs/P2X_7_Rs and Panx1 channels in neurons. Thus, the prevention of HC- and pannexon-mediated microglial activation represents an unexplored strategy to prevent neuronal damage and death during neuroinflammation. Future studies will elucidate the impact of this signaling pathway in neurodegenerative diseases *in vivo*. The findings discussed here strengthen the emerging concept that the unregulated membrane permeability of HCs, pannexons and GJCs contributes to microglial dysfunction. Thus, connexins as well as pannexins represent potential and alternative targets for therapeutic intervention in neuroinflammatory diseases.

## Clinical Relevance

Although the clinical impact of dysfunctional HCs and pannexons in microglia is still unknown, gene mutations and changes in expression or distribution of connexins and pannexins have been implicated in the genesis and progression of different diseases (Penuela et al., 2014; Retamal et al., 2015; Bai, 2016). For instance, germline mutations in genes encoding Cx26, Cx30, Cx31, Cx32 and Cx43 are linked with almost the half of all cases of inherited neurosensory deafness (Martínez et al., 2009), whereas Cx32 and Cx47 mutations are associated with X-linked Charcot–Marie–Tooth (Kleopa et al., 2006) and Pelizaeus–Merzbacher-like (Bugiani et al., 2006) diseases, respectively. In addition, mutations in Cx46 and Cx50 are connected with congenital cataracts (Gong et al., 2007), whereas people with Cx43 mutations show the developmental disorder oculodentodigital dysplasia (ODDD) that in a few cases is accompanied by palmoplantar keratoderma (Paznekas et al., 2009). Along with the diseases associated with connexin gene mutations, there is another group of pathologies that involve changes in the expression, assembly state or localization of connexins and/or pannexins. The loss of gap junctional communication in different human cell-derived tumors arises as the most remarkable example of connexin regulation during pathology (Jiang and Penuela, 2016). Another illustrative example is the relocalization of Cx43 and gap junction modulation that participate in arrhythmias and heart failure (Severs et al., 2008). In the CNS, patients with temporal lobe epilepsy exhibit high levels of Panx1 compared to normal people (Jiang et al., 2013), whereas Cx43, Cx30, Cx36 behave similarly in rodent models of epilepsy (Mylvaganam et al., 2014). In the same context, cortical spreading depression associated to Cx36 and Panx1 dysfunction has been linked to the pathophysiology of migraine with aura (Sarrouilhe et al., 2014). Recently, Cepeda et al. (2015) showed that brain samples from children undergoing Rasmussen encephalitis exhibit an increased expression of Panx1 and Cx36 in Iba-1-positive cells compared to control conditions. These findings put to the forefront the potential therapeutic role that could have connexin and pannexin-derived channels in different neurological disorders.

One of the most interesting challenges in the connexin and pannexin field is to develop molecular strategies to specifically target the expression and/or function of GJCs, HCs and pannexons. Currently, most pharmacological (e.g., drugs and mimetic peptides) and molecular (e.g., siRNAS and KO mice) tools in animals models do not allow the discrimination between GJC vs. HC/pannexon function (Sáez and Leybaert, 2014). However, recent clinical evidence suggests that these channels could be highly exploitable for pharmacological intervention or serve for genetic screening purposes. At clinical level, two major interesting approaches have been used to modulate connexin/pannexin expression or the channels themselves: targeting transcription/translation (e.g., antisense approaches) and peptidomimetic strategies (e.g., Gap26, Gap27, Peptide5, Gap19 and ACT-1). In this context, nowadays there are four well-known companies with human therapeutic focus on connexins and pannexins: CoDa Therapeutics Inc. (USA and New Zeland), FirstString Research (USA), Theranexus (France) and Zealand Pharma (Denmark). A general patent search for “connexin” or “pannexin” at the US Patent and Trade-mark Office website reveals more than 500 and 25 issued, respectively. Different drugs and peptides designed to modulate GJC and/or HCs have shown stronger therapeutic effects in different human pathological conditions including venous leg ulcers, eye inflammation and trauma, as well as glaucoma (Becker et al., 2016). Future studies will open new horizons to unveil the multiple therapeutic opportunities that are behind the function and regulation of connexin and pannexin-based channels.

## Author Contributions

RG-G, VCL and JAO: conceived and designed the major ideas developed in the manuscript, reviewed the literature and designed the tables and figures. JAO: wrote and edited the manuscript. All authors read and approved the final manuscript.

## Funding

This work was supported by Fondo Nacional de Desarrollo Científico y Tecnológico (FONDECYT) grants 11121133 (to JAO) and 1160710 (to JAO) and the Comisión Nacional de Investigación Científica y Tecnológica (CONICYT) and Programa de Investigación Asociativa (PIA) Grant Anillo de Ciencia y Tecnología ACT1411 (to JAO).

## Conflict of Interest Statement

The authors declare that the research was conducted in the absence of any commercial or financial relationships that could be construed as a potential conflict of interest.
